# Imputing missing genotypes: effects of methods and patterns of missing data

**DOI:** 10.1186/1753-6561-5-S7-P61

**Published:** 2011-09-13

**Authors:** Funda Ogut, Fikret Isik, Steven McKeand, Ross Whetten

**Affiliations:** 1 Cooperative Tree Improvement Program Department of Forestry and Environmental Resources, North Carolina State University Raleigh, NC, USA; 2 Cooperative Tree Improvement Program, Department of Forestry and Environmental Resources, North Carolina State University, Raleigh, NC, USA

## 

Costs of high-throughput genotyping have decreased to the point where it appears economically feasible to use molecular genetic marker information in applied breeding programs. Some practical questions remain to be addressed about how best to deal with missing data in the resulting genotype datasets, to minimize the impact of the missing data on the accuracy of breeding value prediction. Data can be missing for two reasons – first, genotyping assay failure is likely for at least some loci in some samples; and second, it may prove economically desirable to invest more resources for high-density genotyping of a few individuals and fewer resources for lower-density genotyping of many individuals [1]. The proportion of missing genotypes may range from less than one percent due to genotyping assay failure, to over 80% if a selective genotyping strategy is used. Many methods for predicting genetic merit of trees using marker genotype data require complete genotype information for mathematical reasons. It is therefore important to use efficient statistical methods to accurately impute missing genotypes. In species with complete reference genome sequences available, the map order of markers and linkage disequilibrium (LD) information can be used to guide imputation of missing genotypes. Completely sequenced reference genomes are available for only two forest tree species, so these methods are not suitable for most forest trees.

Gengler et al. [2] described a method to impute missing genotypes using mixed linear models and BLUP. We determined the effect on accuracy of BLUP estimated breeding values of imputation with different levels (10%, 20%, 40%, 60% and 80%) of missing genotypes. Analyses were conducted both with empirical data (3461 SNP markers in a cloned loblolly pine population of 165 genotypes) and simulated data, using missing data created by random sampling (some loci missing in all individuals) or by structured sampling (all loci missing in some individuals). Simulations were used to examine the effect of family and progeny size, mating design, proportion of missing genotypes, genotyping strategy and the method for imputation on the accuracy of breeding values. Imputed genotypes were obtained using the numerator relationship matrix (the **A** matrix) and solving the mixed model equations of **y = Xb + Mu + e**, where **y** is the vector of gene content predictions, **X** is the design matrix (vector of 1s) for the mean, **M** is the design matrix connecting trees to the gene content vector **y**, **u** is the individual tree effect and **e** is the error variance. The solutions of mixed model equations produce predicted SNP genotypes for trees with missing genotypes. The solutions would be continuous, centered on 1 because the gene content values are 0, 1 or 2.

Imputation of missing genotypes in empirical data from an unbalanced mating design with family sizes ranging from 1 to 35 was more powerful for data with structured missing genotypes at all levels of missing data than for data with random missing genotypes with same proportions of missing data. The accuracy of imputation for 10% and 80% missing genotypes ranged between 0.96 to 0.23 and 0.96 to 0.16 for structured and random missing genotypes in the data, respectively. As the proportion of missing genotypes increased in the data, the power of imputation decreased. With simulation, we found that the imputation was less affected by the distribution of missing genotypes in a balanced mating design with families of equal size. The accuracy of imputation ranged between 0.97 to 0.75 for the 10% and 80% missing genotypes in the data, respectively.
